# Health-Related Quality of Life of Economically Disadvantaged African American Older Adults: Age and Gender Differences

**DOI:** 10.3390/ijerph16091522

**Published:** 2019-04-29

**Authors:** Shervin Assari, James Smith, Mohsen Bazargan

**Affiliations:** 1 Department of Family Medicine, Charles R. Drew University of Medicine and Science, Los Angeles, CA 90095, USA; jamessmith@cdrewu.edu (J.S.); mohsenbazargan@cdrewu.edu (M.B.); 2 Department of Family Medicine, University of California Los Angeles (UCLA), Los Angeles, CA 90095, USA

**Keywords:** race, gender, blacks, African Americans, ethnic groups, age, Health-Related Quality of Life

## Abstract

*Background*: The association between age and health-related quality of life (HRQoL) is still under debate. While some research shows older age is associated with better HRQoL, other studies show no or negative association between age and HRQoL. In addition, while the association between age and HRQoL may depend on race, ethnicity, gender, and their intersections, most previous research on this link has been performed in predominantly White Middle Class. *Objective*: To explore gender differences in the association between age and mental and physical HRQoL in a sample of economically disadvantaged African American (AA) older adults. *Methods*: This cross-sectional survey was conducted in South Los Angeles between 2015 to 2018. A total number of 740 economically disadvantaged AA older adults (age ≥ 55 years) were enrolled in this study, using non-random sampling. This includes 266 AA men and 474 AA women. The independent variable of interest was age. Dependent variables of interest were physical component scores (PCS) and mental component scores (MCS), two main summary scores of the HRQoL, measured using Short Form-12 (SF-12). Gender was the moderator. Socioeconomic status (educational attainment and financial difficulty) were covariates. Linear regression models were used to analyze the data. *Results*: AA women reported worse PCS; however, gender did not impact MCS. In the pooled sample, high age was associated with better PCS and MCS. In the pooled sample, a significant interaction was found between gender and age on PCS, suggesting a stronger effect of age on PCS for AA men than AA women. In gender-stratified models, older age was associated with better PCS for AA men but not AA women. Older age was similarly and positively associated with better MCS for AA men and women. *Conclusions*: There may be some gender differences in the implications of ageing for the physical HRQoL of AA older adults. It is unclear how old age may have a boosting effect on physical HRQoL for AA men but not AA women. Future research should test gender differences in the effect of age on physical health indicators such as chronic disease as well as cognitive processes involved in the evaluation of own’s health in AA men and women.

## 1. Introduction

Health-Related Quality of Life (HRQoL) predicts a wide range of health outcomes, including falls [1], health care use [2], and mortality [2,3,4]. As a result, there has been increasing interest in understanding demographic, behavioral, and social factors that shape HRQoL across various populations [5,6,7]. There is also a particular interest in understanding the factors that can explain decline and those that can be used to enhance HRQoL of aging populations, particularly in demographic and social groups in which HRQoL is low [8,9,10,11,12,13]. Given the consistent disparities across multiple domains of health, African Americans (AAs) report worse HRQoL than Whites [14,15,16,17,18,19]. As a result, it is important to describe the subgroups of AA older adults who report high HRQoL.

There is no agreement on how older age influences HRQoL [20,21,22]. While some research suggesting that older age is associated with better HRQoL, other studies report no effect or worse HRQoL of older adults [22]. Most of the research on the association between age and HRQoL has used predominantly White samples, with less knowledge being available on other racial and ethnic groups such as AAs [21,22,23]. Overall, few studies have investigated HRQoL in AA older adults [11,12]. As the meaning and determinants of subjective aspects of health vary across populations [24,25,26,27], measures such as HRQoL may have different meanings across social groups, and the same HRQoL measures may be differently impacted by same demographic and social and health factors [28,29,30]. One of the factors that may differently impact HRQoL of various populations is age, particularly because age-related changes in life conditions, health, socioeconomic status, and social relations differ across various populations [28,29,30].

For example, falls may be a mechanism that links age to HRQoL [1]. In a study, AAs had better mental HRQoL than Hispanics, suggesting some resilience under adversity among AAs with chronic conditions such as cancer [31,32]. Still, most of the studies are on a particular sample with a chronic condition or disease [19,33]. As most of this research has used disease specific HRQoL which is not generalizable to all general population [34]. More research is needed on the intersections of age, gender, and race on HRQoL of populations.

Some research suggests that not only race and ethnicity [35] but also gender may alter correlates of HRQoL across populations [36,37,38,39]. The effect of gender on shaping subjective aspects of health such as HRQoL differs by location, race, ethnicity, and culture [38,40,41]. We know more about how the meaning and correlates of subjective health measures such as HRQoL and self-rated health (SRH) in Whites than in other racial and ethnic groups.

In line with the global trends, the American population is undergoing an unprecedented ageing process, which is accompanied with and increased life expectancy and presence of multimorbidity. Increased life expectancy combined with an increase in prevalence of non-communicable diseases increases the number of years that the population of older adults live [42]. As a result, there is particularly important to understand how process of ageing is associated with a change in HRQoL [14]. In such a context, maintaining high HRQOl, becomes a marker of healthy ageing, an important objective for policy-makers. Healthy ageing, defined as “the process of developing and maintaining the functional ability that enables well-being in older age” [42], relies on high HRQoL. As a result, understanding HRQoL in older adults, and the effect of age on HRQoL is of primary relevance. Most studies addressing determinants of HRQoL of populations generally focus on patients with a chronic disease. Research addressing gender differences in the impact of age on HRQoL of economically disadvantaged AA older adults is, however, almost lacking.

### Aims

To better understand whether gender alters meanings and correlates of subjective health measures in low-income AA older adults [43,44,45,46], this study compared economically disadvantaged AA men and AA women for the association between age and physical and mental HRQoL. In line with other studies that show major gender differences in the meaning of subjective health measures [47,48], we expected differential effects of age on physical and mental HRQoL between older AA men and AA women. We did not have any specific hypothesis regarding stronger or weaker effects of age in a particular gender. This paper extends the existing literature from other populations to economically disadvantaged AAs in an under-resourced urban area.

## 2. Methods

### 2.1. Design & Setting

This was a cross-sectional survey of economically disadvantaged AA older adults in South Los Angeles. The study was performed between 2015 and 2018.

### 2.2. Process and Data Collection 

The data collection included structured face-to-face interviews and a comprehensive assessment of medications. The interview collected data on demographic factors (age and gender), SES (educational attainment and financial difficulty), and HRQoL.

### 2.3. Participants

The study used a non-random sampling strategy to recruit AA older adults from economically disadvantaged areas in South Los Angeles. Using a convenience sampling, participants were eligible if they were AA or Black, were 55 years or older, could complete an interview in English, and were living in SPA6. Institutionalized participants were excluded from the study. Other exclusion criteria included being enrolled to any clinical trial. This sampling resulted in 740 economically disadvantaged AAs aged 55 years and older. 

### 2.4. Comparability of Our Sample

Demographics of our participants were comparable to those of AA older adults in South Los Angeles. In our sample, only 35% had a high school diploma. With a similar pattern, in the California Health Interview Survey (CHIS) data, 37% of AA older adults in South Los Angeles had a high school diploma. Regarding the health status of our participants, about one third described their SRH as fair or poor. Again, similarly, CHIS data show that 38% of AA older adults with the same age group living in South Los Angeles report fair or poor SRH [49]. 

### 2.5. Measures

#### 2.5.1. Outcome Variables 

The HRQoL was measured using SF-12v2, which is a 12-item measure. This measure generates two summary scores and eight sub-domains (or subscales). Summary scores include the Physical Component Summary (PCS) and the Mental Component Summary (MCS) scores. Bodily Pain (BP), General Health (GH), Vitality (VT), and Social Functioning (SF) with one item each; and physical Functioning (PF), Mental Health (MH), Role Physical (RP), and Role Emotional (RE) domains, each with two items. To score the SF-12v2, we followed the method proposed by the original authors. The summary scores are calculated from z-scores of the 8 subscales. All scales contribute to the scorings of PCS and MCS, using weights from principal component analysis on the SF-36 scales. The norm-based scoring that is commonly used for SF-12v2 produces scores with a mean of 50 and a standard deviation of 10 for the US population. A higher score indicates better HRQoL [50,51,52,53,54,55,56,57,58,59,60].

#### 2.5.2. Predictor Variable

Age was treated as an interval variable. 

#### 2.5.3. Confounders

Educational attainment and financial difficulty were the SES covariates in this study. Educational attainment was operationalized as an interval level variable (years of schooling). Higher scores indicated more years of education. Financial difficulty was measured using three items. These items asked about the frequency of not having enough money to afford (1) food, (2) clothing, and (3) paying bills. Items were on a 5-level response scale ranging from 1 (never) to 5 (always). A total “financial difficulty” score was calculated, with a score ranging from 3 to 15. A high score was indicative of more financial difficulty. These items are consistent with Pearlin’s list of chronic financial difficulties of low SES individuals [61]. (Cronbach alpha = 0.92).

#### 2.5.4. Moderator

Gender, the effect modifier, was treated as a dichotomous variable (1 = female; 0 = male).

### 2.6. Statistical Analysis

Data analysis was performed in SPSS 23.0 (IBM Corporation, Armonk, NY, USA). For descriptive purposes, means, standard deviation (SD), and frequencies (%) were reported, both in the pooled sample and by gender. We used the independent samples t test and the Chi-squared test to compare our study variables between AA men and AA women. We also ran the Pearson correlation test to explore correlations between study variables in the pooled sample and by gender. For multivariable analysis, we used eight linear regression models, four for PCS and four for MCS as the outcome. In our models, age was the main independent variable, either PCS or MCS was the main outcome, SES (educational attainment and financial difficulty) were the covariates, and gender was the moderator. From linear regression models, b (regression coefficient), standard errors (SE), *t* Values, and *p* Values were reported. We ruled out multicollinearity between our predictors. We also tested the homoscedasticity of our outcomes, defined as having equal statistical variances. We replicated our model for self-rated health as outcome (Appendix A).

### 2.7. Institutional Review Board (IRB)

The study protocol was approved by the Institutional Review Board (IRB) of the Charles R. Drew University of Medicine and Science (CDU), Los Angeles. All participants signed a written informed consent before being enrolled in the study. Participants received a financial incentive.

## 3. Results

### 3.1. Descriptive Statistics

A total number of 740 economically disadvantaged AA older adults who were at least 55 years old entered to this study. From this number, 474 were AA women and 266 were AA men. Average age of the participants was 71.73 (SD = 8.37). Mean educational attainment of this sample was 12.74 (SD = 2.24). On average, their PCS was considerably lower than average US population (mean 40.29, SD = 12.22), however, their MCS was about average US population (52.28 ± 10.93).

Table 1 describes the sample overall and also by gender. This table also compares AA men and AA women for age, educational attainment, financial difficulties, PCS, and MCS. In this study, AA men and AA women differed in age, educational attainment, and PCS and MCS. Regarding age, AA men were younger than AA women. AA women had higher educational attainment than AA men. While PCS was better in AA men than AA women, MCS was better in AA women than AA men. 

### 3.2. Bivariate Correlations

Table 2 shows three correlation matrices between the study variables in the overall sample and for AA men and AA women. As this table shows, in the pooled sample, PCS and MCS were not correlated in the pooled sample, AA men, or AA women. In the pooled sample, male gender was positively and negatively correlated with PCS and MCS, respectively. High educational attainment was associated with better MCS but not PCS in the pooled sample. High educational attainment was associated with better MCS for AA women but not AA men. Older age was correlated with better PCS and MCS in the pooled sample as well as AA men and AA women. 

### 3.3. Linear Regressions in the Pooled Sample

Table 3 shows the results of four linear regression models in the overall sample, with two models having PCS as the outcome and two models having MCS as the outcome. As this table shows, in the pooled sample, age was positively associated with PCS and MCS. 

In the pooled sample, a gender-by-age interaction was found in the model with PCS as the outcome, suggesting that the positive association between age and PCS is stronger for AA men than AA women. In the pooled sample, gender and age did not interact on MCS as the outcome, suggesting that the positive association between age and MCS does not differ between AA men and AA women.

### 3.4. Linear Regressions in AA Men and AA Women

Table 4 shows the results of four gender-specific linear regression models. Two of these models have PCS as the outcome and two of the linear regression models have MCS as the outcome. As this table shows, age was positively associated with MCS in both AA men and AA women. Age was positively associated with PCS in AA men but not in AA women.

## 4. Discussion

In a sample of economically disadvantaged AA older adults in LA, the current study showed two findings. First, age was positively, not negatively, associated with HRQoL in the pooled sample. This pattern was consistent for PCS and MCS and could be replicated for SRH, another well accepted subjective measure of health (Appendix A). Second, this study documented a gender difference in the association between age and physical HRQoL. Older age was associated with better physical HRQoL in AA women but not AA men. For both AA men or AA women, older age was associated with better mental HRQoL. 

Our first finding on the positive association of age with physical and mental HRQoL, which could be replicated for SRH, is similar to some, but not all, studies. In a study that involved 247 older adults composed of 154 Whites, 90 AAs, and 3 Asians, participants reported an improvement in the HRQOL over a two-year follow up period. The study showed that the AAs had greater improvements in mental HRQoL compared to Whites [62]. 

Overall, age has shown inconsistent effects on HRQoL across studies [63]. Even within a same study, age may have different effects on domains of HRQoL of one population [64]. There are also studies on HRQoL of older AAs which has not included age as a covariate [65]. Some studies have suggested worse HRQoL in older adults [66]. In a study on patients with CMC(s), older age did not result in poor HRQoL across all domains. In fact, older age was associated with better general health perception, suggesting that the general aspect of HRQoL may improve in the elderly, which might be due to changes in coping abilities or expectations. For older adults, HRQoL may reflect a general sense of well-being and life satisfaction rather than physical function [67]. Some research has shown that for older adults, social functioning becomes a more important part of HRQoL than physical health and physical function [68]. Thus, meaning and interpretation of HRQol is unique to the elderly population [69,70,71,72,73,74,75,76,77,78,79,80]. Group differences in the meaning of SRH is commonly shown [62,63,64,65,66,73].

The observed positive associations between age and PCS/MCS in this study may also be due to the mortality cross-over that happens in AAs, compared to Whites [64,65,66,67,68,69,70,71,72,73,74,75,76,77,78,79,80]. Due to high mortality before age 65, AA individuals who survive until 65 may be hardier than their peers who did not had the chance to survive to such age. As a result, PCS and MCS of AA individuals who have reached such advanced age may be high because they are a selected healthy group [78,79,80]. 

Among studies on population differences in determinants of subjective health, there are fewer studies on HRQoL and more studies on SRH. In a recent study in AA adults, for women, SRH was found to operate like a sponge; it absorbed more affective and contextual information compared to AA men [63]. Other studies have also found some related findings. In a study in AA individuals with diabetes, SRH reflected glucose control for AA men but not AA women [47]. In another study of people with diabetes, worse glycemic control (Higher HbA1c) was associated with worse level of SRH in males and females only when all age groups were combined. However, in younger people, the very same association was stronger for women than men, probably due to diabetes-related worries as a result of high HbA1c [67]. Thus, various studies have documented gender differences in the meaning of subjective health outcomes such as gender, race, and their intersections.

Gender differences exist in subjective measures of well-being and health (e.g., HRQoL and SRH). In mainly White samples, poor SRH predicted risk of mortality of men better than women [48,68]. In one of the studies, the author argued that in women, SRH may reflect more contextual and affective information, whereas for men, the main determinant of SRH is CMC [48]. In another study, gender difference in the predictive power of poor SRH on the risk of mortality was attenuated by controlling for co-morbid conditions, suggesting that CMC is one of the reasons SRH better predicts mortality of men than women [68]. Most of this literature, however, is mainly on samples that are predominantly White [48]. The main contribution of this study is to extend this literature to low-income AAs.

There are also studies that do not confirm major gender differences in meanings of SRH. In another study spanning 12 years from the Health and Retirement Study (HRS), males and females were compared for trajectories and determinants of SRH. The study, which was mainly composed of Whites, did not show gender differences in the SRH levels at baseline; however, SRH declined faster for men than women over time. Onset of development of CMC, health behaviors such as smoking, and rate of retirement explained the observed gender difference in trajectory of SRH over time [69].

In a study that used the 2002–2015 National Health Interview Survey (NHIS) data, ordered logistic regression models were applied to predict SRH as a function of two dozen health conditions including CMC, physical symptoms, mental health, function, health care use, and health behaviors by gender. The study found almost no evidence supporting the sponge hypothesis. The study failed to show systematic gender variation in the structure of SRH. The study showed that men and women use a wide-range of health-related frames of reference, mostly in a similar way, to make a judgment regarding their own health. The following gender differences were observed: at mid-life and older ages, men have a higher tendency than women to weigh physical functioning and negative health behaviors as a factor in SRH. The study suggested that only through mid-adulthood, women report worse SRH than men. This pattern is reversed in older ages. The study also showed that the female disadvantage in SRH is fully due to SES differences. The study argued that SRH can be used to measure gender differences in health [70]. A study in veterans also did not find major gender differences; however, exposure to warfare was more predictive of SRH for men than for women [71].

Gender, race/ethnicity, SES, and their intersections change how subjective measures such as SRH and HRQoL reflect health problems such as CMC, depression, and mortality. In other words, poor status in subjective measures do not have the same meaning across groups [62,63,64,65,66]. For example, education and income improves subjective health of Whites but not AAs [72,73,74]. At the same time, SRH predicted risk of mortality of Whites but not AAs [75,76]. This is because SRH does not reflect the same aspects of health for ethnic groups [77,78] and is not the same across countries [38,41,79,80]. In the Fragile Families and Child Well-being Study which followed 2407 AAs, 1354 Hispanic Whites and 894 non-Hispanic Whites for five years for changes in SRH, in all ethnic groups, anxiety and drinking problem were predictive of poor SRH at baseline and over time. However, the study documented cross-ethnic variation in the combined (additive) effects of general anxiety disorder (GAD) and major depressive disorder (MDD) on SRH. For AAs, MDD and GAD both predicted a worse trajectory of SRH over time. For non-Hispanic Whites, MDD predicted a worse baseline SRH while GAD predicted better SRH at baseline and over time. For Hispanic Whites, GAD predicted a worse trajectory of SRH; however, MDD was not linked to SRH [62]. In another cross-sectional study borrowing data from the National Survey of American Life 2003, 3570 AAs and 1621 Caribbean Blacks were entered. For AAs, GAD and MDD had independent (i.e., separate) effects on mental SRH. For Caribbean Blacks, however, MDD but not GAD independently affected mental SRH. When the joint (e.g., combined) effects of GAD and MDD were explored, GAD but not MDD impacted poor mental SRH [65]. 

### Limitations

study, causal associations are not plausible. This study did not control several factors such as income, history of psychiatric disorders, chronic diseases. Unbalanced sample size between AA men and AA women may have resulted in differential statistical power between AA men and AA women. This is not a concern, as we found stronger association in the smaller group (with lower statistical power). In addition, with the convenience sampling in that area and excluded institutionalized participants, the results may have over-estimated the HRQoL or underestimated the effects of covariates on the outcome variables. In addition, we only focused on physical and mental HRQoL. Other researchers may also focus on subscales of HRQoL, which were not of interest here. We can also not rule out the possibility that gender differences in response styles result in the observed gender differences in HRQoL. Our study failed to explain possible self-report differences on the data collection. These limitations should be addressed in future studies examining older AA adults’ HRQoL. Despite these limitations, this study contributes to the literature on the intersectional effects of race, gender, and age on populations’ HRQoL. 

This study is unable to make a distinction between age- and cohort- differences. Although, as all participants were at least 55 years old, participants were at most one generation different from each other. Particularly in studies that use a cross-sectional design, the association between age and HRQoL may be confounded with cohort effect. Thus, the results may not all be due to aging, but some of it may be due to variations across generations. This issue can be studied in future studies with multiple observations over time. 

## 5. Conclusions

In summation, our findings suggest that among economically disadvantaged AA older adults, age is positively associated with physical and mental HRQoL. Low-income older AA men and women, however, differ in the association of age and physical HRQoL. Age may have a stronger effect on physical HRQoL for economically disadvantaged older AA men than for AA women. The results invite researchers to study how age-related changes in HRQoL differ between AA older men and women.

## Figures and Tables

**Table 1:** Descriptive Statistics in the pooled sample as well as African American men and women.

	All *n* = 740		African American Men *n* = 262		African American Women *n* = 474	
	Mean	SD	Mean	SD	Mean	SD
Age (Years) *	71.73	8.37	70.79	8.32	72.26	8.36
Educational Attainment (Years) *	12.74	2.24	12.42	2.51	12.93	2.06
Financial Difficulty (3–15) ^#^	9.18	5.64	9.70	6.29	8.88	5.23
PCS (0–100) *	40.29	12.22	42.12	12.16	39.25	12.14
MCS (0–100) *	52.28	10.93	51.00	10.68	53.00	11.01

MCS: Mental Component Score; PCS: Physical Component Score; SD: Standard Deviation. ^#^ *p* < 0.1, * *p* < 0.05 (Independent sample *t* test).

**Table 2:** Bivariate correlation matrices in the pooled sample and by gender.

	1	2	3	4	5
**All**					
1 Age (Years)	1.00	−0.18 **	−0.31 **	0.17 **	0.18 **
2 Education (Years)		1.00	−0.08 *	0.00	0.11 **
3 Financial Difficulty			1.00	−0.32 **	−0.32 **
4 PCS				1.00	−0.02
5 MCS					1.00
**AA Women**					
1 Age (Years)	1.00	−0.25 **	−0.32 **	0.31 **	0.20 **
2 Education (Years)		1.00	−0.14 *	−0.01	0.13 *
3 Financial Difficulty			1.00	−0.39 **	−0.35 **
4 PCS				1.00	0.07
5 MCS					1.00
**AA Men**					
1 Age (Years)	1.00	−0.16 **	−0.29 **	0.11 *	0.16 **
2 Education (Years)		1.00	−0.01	0.02	0.08
3 Financial Difficulty			1.00	−0.30 **	−0.30 **
4 PCS				1.00	−0.06
5 MCS					1.00

MCS: Mental Component Score; PCS: Physical Component Score. * *p* < 0.05, ** *p* < 0.01.

**Table 3:** Summary of linear regressions with PCS and MCS as the outcome.

	PCS						MCS					
	b	SE	95% CI		*t*	*p*	b	SE	95% CI		*t*	*p*
			**Model 1**						**Model 1**			
Gender (Male)	3.58	0.89	1.84	5.33	4.03	0.000	−1.17	0.80	−2.73	0.40	−1.47	0.143
Age (Years)	0.13	0.05	0.02	0.23	2.32	0.021	0.15	0.05	0.05	0.25	3.08	0.002
Educational Attainment (Years)	0.03	0.19	−0.36	0.41	0.14	0.891	0.52	0.18	0.18	0.87	3.00	0.003
Financial difficulty	−0.66	0.08	−0.82	−0.51	−8.34	0.000	−0.51	0.07	−0.65	−0.37	−7.21	0.000
			**Model 2**						**Model 2**			
Gender (Male)	−14.45	7.58	−29.33	0.42	−1.91	0.057	−2.78	6.81	−16.16	10.60	−0.41	0.683
Age (Years)	0.04	0.07	−0.09	0.17	0.60	0.546	0.14	0.06	0.03	0.26	2.42	0.016
Educational Attainment (Years)	0.06	0.19	−0.32	0.44	0.30	0.762	0.53	0.18	0.18	0.87	3.01	0.003
Financial difficulty	−0.65	0.08	−0.81	−0.50	−8.23	0.000	−0.51	0.07	−0.65	−0.37	−7.18	0.000
Gender (Male) × Age (Years)	0.25	0.11	0.05	0.46	2.40	0.017	0.02	0.09	−0.16	0.21	0.24	0.812

SE: Standard Error, CI: Confidence Interval; MCS: Mental Component Score; PCS: Physical Component Score.

**Table 4:** Summary of linear regressions with PCS and MCS as the outcomes by gender.

	PCS						MCS					
	b	SE	95% CI		*t*	*p*	b	SE	95% CI		*t*	*p*
**Model 3 (Females)**												
Age (Years)	0.04	0.07	−0.10	0.17	0.56	0.577	0.14	0.06	0.02	0.26	2.26	0.025
Education (Years)	0.14	0.27	−0.38	0.66	0.53	0.598	0.54	0.24	0.07	1.00	2.25	0.025
Financial difficulty	−0.68	0.11	−0.89	−0.47	−6.28	0.000	−0.54	0.10	−0.73	−0.35	−5.55	0.000
**Model 4 (Males)**												
Age (Years)	0.29	0.09	0.11	0.47	3.23	0.001	0.17	0.08	0.01	0.33	2.12	0.035
Education (Years)	−0.03	0.29	−0.60	0.53	−0.11	0.915	0.53	0.26	0.02	1.04	2.04	0.042
Financial difficulty	−0.63	0.12	−0.86	−0.40	−5.37	0.000	−0.48	0.11	−0.69	−0.27	−4.55	0.000

SE: Standard Error; CI: Confidence Interval; MCS: Mental Component Score; PCS: Physical Component Score.

## Appendix A

**Table A1:** Associations between age and self-rated health (SRH) in the pooled sample.

	B	S.E.	Exp(B)	Lower	Upper	*t*	*p*
**Poor SRH**							
Gender (Male)	−0.18	0.19	0.84	0.57	1.22	0.85	0.357
Age (Years)	−0.05	0.01	0.95	0.93	0.98	12.08	0.001
Educational Attainment (Years)	−0.06	0.04	0.94	0.87	1.02	2.48	0.115
Financial Difficulty	0.05	0.02	1.05	1.02	1.09	8.70	0.003
Constant	3.40	1.30	29.94			6.84	0.009
**SRH (1–5)**							
Gender (Male)	−0.19	0.09	−0.09	−0.36	−0.02	−2.21	0.027
Age (Years)	−0.02	0.01	−0.15	−0.03	−0.01	−3.69	0.000
Educational Attainment (Years)	−0.02	0.02	−0.05	−0.06	0.01	−1.28	0.200
Financial Difficulty	0.03	0.01	0.16	0.02	0.05	3.85	0.000
Constant	4.79	0.57		3.68	5.91	8.47	0.000

SRH: Self-Rated Health.
